# The DdD protein confers intracellular and extracellular immunity to the leaderless bacteriocin enterocin DD14

**DOI:** 10.1093/pnasnexus/pgag192

**Published:** 2026-06-12

**Authors:** Clémence Cochard, Adrián Pérez-Ramos, Mari Luz Mohedano, Mario García de Lacoba, Paloma López, Djamel Drider

**Affiliations:** Unité Mixte de Recherche (UMR) Transfrontalière BioEcoAgro INRAE 1158, Université de Lille, 59000 Lille, France; Unité Mixte de Recherche (UMR) Transfrontalière BioEcoAgro INRAE 1158, Université de Lille, 59000 Lille, France; Departamento de Biotecnología, Centro de Investigaciones Biológicas Margarita Salas (CIB, CSIC), 28040 Madrid, Spain; Departamento de Bioinformatics y Biostatistics, Centro de Investigaciones Biológicas Margarita Salas (CIB, CSIC), 28040 Madrid, Spain; Departamento de Biotecnología, Centro de Investigaciones Biológicas Margarita Salas (CIB, CSIC), 28040 Madrid, Spain; Unité Mixte de Recherche (UMR) Transfrontalière BioEcoAgro INRAE 1158, Université de Lille, 59000 Lille, France

**Keywords:** leaderless bacteriocins, enterocin 14, self-protection, immunity, DdD protein

## Abstract

Leaderless bacteriocins (LBBs) are ribosomally synthesized antimicrobial peptides that differ from classical bacteriocins because they are produced in their active form without leader peptides. This raises the key biological question of how producing strains protect themselves from their own bacteriocins during synthesis and prior to secretion. Enterocin DD14 (EntDD14) is a two-peptide LBB produced by *Enterococcus faecalis* 14. It has antibacterial and antiviral activities, along with immunomodulatory properties. The *EntDD14* gene cluster contains 10 open-reading frames, most of which are involved in production, transport, or immunity. However, the function of the products of the *ddC* and *ddD* genes remains unknown. Here, we present the first functional characterization of the DdD protein. Although in silico analyses predicted that DdD is a membrane protein with multiple transmembrane domains and a prominent cytoplasmic helix, no homologs with defined functions were identified. Genetic analyses revealed that deletion of the *ddD* gene was only feasible in a *Δbac* background lacking the *ddA* and *ddB* bacteriocin structural genes. This indicates that lethality provoked by *ddD* deletion is bacteriocin dependent. Functional assays revealed that DdD is not necessary for EntDD14 synthesis and export but is essential for self-immunity. The *ΔddD* mutants exhibited reduced resistance to EntDD14, when it was provided extracellularly, and impaired growth when it was produced intracellularly. Expression of *ddD* in a multicopy plasmid restored full resistance, confirming its role in self-protection. Thus, DdD emerges as a novel immunity determinant of the EntDD14 system, expanding the repertoire of accessory proteins required for LBB self-resistance.

Significance statement Bacteriocin-producing bacteria must protect themselves from their own antimicrobial peptides; yet, the immunity mechanisms of leaderless bacteriocins (LBBs) remain poorly understood. Enterocin DD14 (EntDD14), produced by *Enterococcus faecalis* 14, is a potent multifunctional LBB that requires robust self-protection during active synthesis. In this study, we identify the DdD protein as a crucial immunity factor that defends against both intracellular and extracellular EntDD14. These findings refine the current model of LBB immunity by highlighting the essential role of accessory proteins in cooperation with transporters. Understanding these mechanisms not only deepens our knowledge of *E. faecalis* biology but also enables the rational design of producer strains with controlled bacteriocin expression, preventing self-toxicity and unintended ecological effects. Furthermore, this insight supports the development of safer probiotics, therapeutics, and food biopreservation strategies.

## Introduction

Antibacterial peptides (AMPs) are a widespread defense strategy found in all domains of life, functioning to prevent infection or mediate microbial competition. AMPs typically act rapidly and often have a broad spectrum of efficacy, which reduces the likelihood of resistance development. Bacteriocins, in particular, are attracting increasing interest due to the rise of antibiotic-resistant pathogens (1). Bacteriocins are ribosomally synthesized peptides that are either narrow spectrum, targeting closely related species, or broad spectrum, acting across genera (2–4). Their mechanisms of action are diverse and include the formation of membrane pores, the inhibition of peptidoglycan or protein synthesis, and the disruption of DNA replication or transcription (1, 5–8). Classical bacteriocins are synthesized as inactive precursors with N-terminal leader sequences that are cleaved upon secretion. Genes involved in the synthesis, modification, and secretion of bacteriocins are generally organized into clusters that encode structural, modification, transport, and immunity proteins. These clusters may be located on plasmids, transposons, or chromosomal regions (9). The export of bacteriocins is usually mediated by an ATP-binding cassette (ABC) transporter or, less commonly, the general secretion (Sec) pathway (10–15). Some bacteriocins require specific secretion systems (16–19). Additionally, accessory proteins may facilitate secretion, leader peptide cleavage, and posttranslational modifications (PTMs) (20–23).

Bacteriocin-producing bacteria also encode specific immunity proteins that protect the producer cell from its own bacteriocins. There are two main protection systems: dedicated immunity proteins and specialized ABC transporters that expel bacteriocins from the producer cell (24–28). These systems can operate independently or together (27, 29–31). Despite their low-sequence similarity, immunity proteins use conserved mechanisms, such as interfering with bacteriocin–receptor interactions via competitive antagonism, blocking membrane pores by sequestering structural components, and inactivating bacteriocins via proteolytic degradation (32–38). Although they are highly effective, immunity proteins are usually highly specific and rarely provide protection against other bacteriocins. However, exceptions have been reported (27, 39–47).

The classical model of bacteriocin synthesis involves producing inactive prepeptides containing an N-terminal leader sequence. However, a subset of bacteriocins, known as leaderless bacteriocins (LBBs), deviate from this paradigm by being synthesized directly in their active form (48). Despite lacking PTM, LBBs exhibit considerable structural diversity, ranging from one to six peptides of 27 to 70 residues (49). Two main modes of action have been described: pore formation, as in lacticin Q and generalized membrane permeabilization, as in aureocin A53 (50–52). Because LBBs accumulate in the cytoplasm in their active form prior to secretion, determining how the producer cells avoid self-destruction remains to be elucidated (48). The immune systems of LBB are poorly understood; in most cases, only candidate proteins have been identified. The most extensively studied system involves an ABC-type multidrug resistance transporter (24, 50, 53), which is thought to contribute to secretion and immunity by lowering bacteriocin concentrations in the cytoplasmic membrane via active efflux (54, 55). However, in some cases, the transporter alone does not provide enough protection. Additional proteins are needed to achieve full immunity (56). Other studies, however, suggest that the ABC transporter is exclusively involved in secretion and that immunity depends entirely on an immunity protein (57).

Enterococci are members of the lactic acid bacteria group and prolific producers of broad-spectrum antimicrobial bacteriocins known as enterocins. These bacteriocins inhibit several foodborne pathogens (58, 59). Enterocin DD14 (EntDD14), produced by *Enterococcus faecalis* 14, a strain originally isolated from meconium (60) has emerged as a leaderless, two-peptide bacteriocin model possessing antibacterial, antiviral, and immunomodulatory properties (61–64). EntDD14 is composed of two peptides (EntDD14A and EntDD14B), whose production is encoded within the EntDD14 gene cluster. The cluster contains 10 open-reading frames (ORFs), designated *ddA–J,* which are organized into two operons (65). The first operon encodes only the two bacteriocin structural genes (*ddA* and *ddB*), while the second operon is thought to be involved in peptide transport and self-immunity. The transport of EntDD14 relies on two distinct systems. The primary system consists of two Pleckstrin-Homology-Domain (PHD)-containing proteins: DdE and DdF (15, 18). The secondary system corresponds to the canonical ABC transporter encoded by *ddGHIJ* (65). This ABC transporter contributes to self-immunity by conferring resistance to EntDD14, when it is supplied from outside (15). However, the mechanisms that protect the producer cell from the newly synthesized, but not yet secreted, bacteriocin are still unknown.

The functions of most of the genes in the two operons responsible for EntDD14 synthesis have been elucidated. However, the roles of *ddC* and *ddD* remain unclear. This study investigated *ddD* at the molecular level to clarify its contribution using genetic evidence derived from constructing several mutant strains. Attempts to delete the *ddD* coding sequence in the wild-type strain were unsuccessful. However, deletion was feasible in the *Δbac* background, when complementation of *ddAB* was performed. The *ΔddD* mutant in the presence of the *ddAB* operon had no defects in the production or secretion of EntDD14; however, it exhibited significantly reduced growth compared with the complemented *ddD* intact strain. Furthermore, deletion of *ddD* decreased resistance to exogenous EntDD14, highlighting its essential role in the self-immunity system of the producer bacterium.

## Results

### In silico characterization of the DdD protein

**Figure 1:**
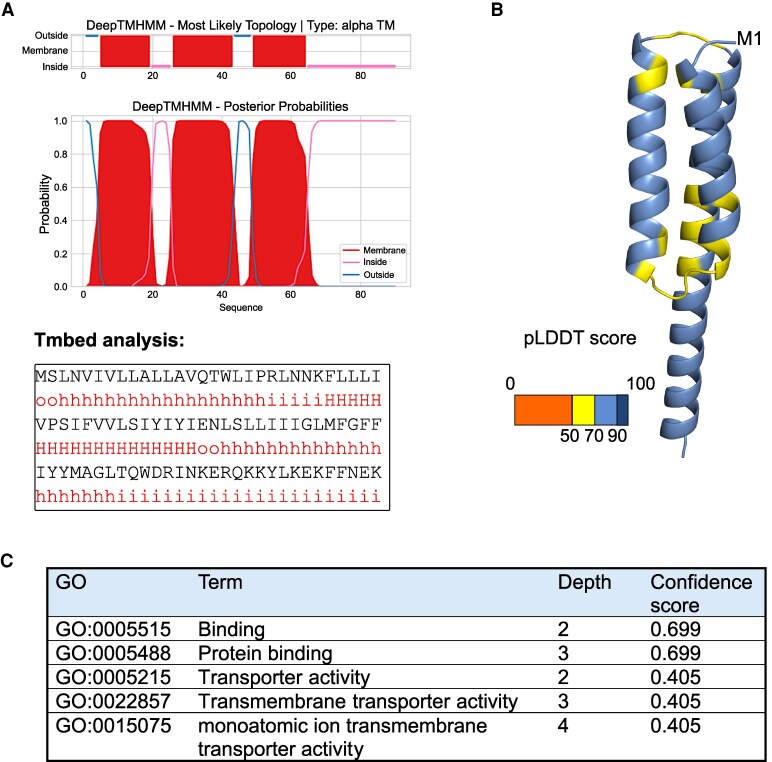
In silico prediction of the structure and function of DdD. A) The predicted transmembrane topology of the DdD sequence was determined using the DeepTMHMM tool (top) and Tmebd (bottom). In the DeepTMHMM analysis, red bars indicate transmembrane domains; pink lines represent intracellular loops; and blue lines represent extracellular loops. The *y*-axis shows the prediction probability, and the *x*-axis corresponds to the aa sequence position. In the TMbed analysis, the DdD aa sequence is shown in black, and the predicted topology is shown in red as follows: o, nontransmembrane outside; i, nontransmembrane inside; H, transmembrane alpha helix (IN → OUT orientation); h, transmembrane alpha helix (OUT → IN orientation). B) The tertiary structure of DdD predicted by AF3 is shown and defined by a color-coded scheme. pLDDT stands for the predicted local distance difference test, which is a per-residue confidence score calculated (between 0 and 100). C) The GO-term enrichment analysis. The top five predicted GO terms in molecular function and their assigned scores are displayed. GO analysis was performed using NetGO 4.0 (score >0.6 for GO terms of high confidence).

To gain initial insight into the molecular features of DdD, we performed structural and sequence-based bioinformatic analyses (Fig. 1). Prediction with DeepTMHMM revealed that DdD is a membrane-associated protein with three transmembrane domains (Fig. 1A). Moreover, to support the DeepTMHMM prediction of the transmembrane orientation of the AF3 DdD model, the Tmbed program was used. This is a machine learning protein language model that predicts the transmembrane location of each residue (Fig. 1A). However, a search of the Protein Data Bank (PDB) did not reveal any DdD homologs with experimentally determined structures. Therefore, AlphaFold 3 (AF3) was employed to predict the structure of DdD. The top-ranked structural model achieved a good confidence score (average pLDDT score = 70.2, in the corresponding range for confident models) and revealed that DdD structure consists of three α-helices, one of which is considerably longer than the others (Fig. 1B). The longest helix is predicted to extend into the cytoplasm, forming a substantial cytoplasmic domain, while only small regions are exposed to the extracellular environment. To investigate possible functions further, gene ontology (GO) term prediction was performed using NetGO 4.0 (Fig. 1C). The top five predicted terms were associated with proteins involved in binding or transport mechanisms. However, none were directly connected with bacteriocin-related processes. Consistently, no functional domains were predicted using InterPro or CD-Search (NCBI). Additional BLASTP searches confirmed the absence of significant sequence homology with characterized proteins. However, strong homology was observed with proteins from *E. faecalis*, *Enterococcus lactis*, *Enterococcus faecium*, and *Enterococcus cecorum*. These included L50F, which is part of the enterocin L50 synthesis cluster. This bacteriocin, synthesized by *E. faecium* L50, is closely related to EntDD14, with 73% amino acid (aa) identity (Fig. S1) among them. Furthermore, a potential role of this protein in transport has been hypothesized (66). Additionally, the aa sequence of DdD was analyzed using the D-I-TASSER program, which uses deep learning to predict three-dimensional (3D) protein structures by assembling fragments from threading templates and performing iterative structural refinement. The predicted secondary structure of DdD was consistent with the structure obtained using AF3, which supports the former model (Fig. 1B). More importantly, D-I-TASSER identified several proteins with significantly similar 3D structures to that of DdD. The closest homolog corresponded to a protein involved in membrane transport, while the others appeared to interact with bacterial toxins (Table 1). Taken together, these results suggest that the DdD protein is a membrane protein involved in the binding and/or transport of EntDD14.

**Table 1:** Proteins structurally similar to DdE and DdF detected with the D-I-TASSER program.

PDB	TM score^a^	Characteristic	Role	Ref.
6M6ZA	0.84	De novo protein	Transmembrane nanopore	(67)
3v0aB4/4zktB	0.84	Clostridial nontoxic nonhemagglutinin protein	Part of toxin progenitor complex	(68, 69)
3vuoa4	0.83	Neurotoxin associating protein	Part of toxin progenitor complex	(70)
5bqnA2	0.72	Fragment of protein	Hydrolase/translocation	(71)

^a^Translational modification (TM) score assesses the topological similarity of protein structures. Values are between 0 and 1. A score >0.5 indicates generally the same fold in SCOP (structural classification of proteins)/CATH (class, architecture, topology, homology).

### DdD is not implicated in EntDD14 synthesis

**Figure 2:**
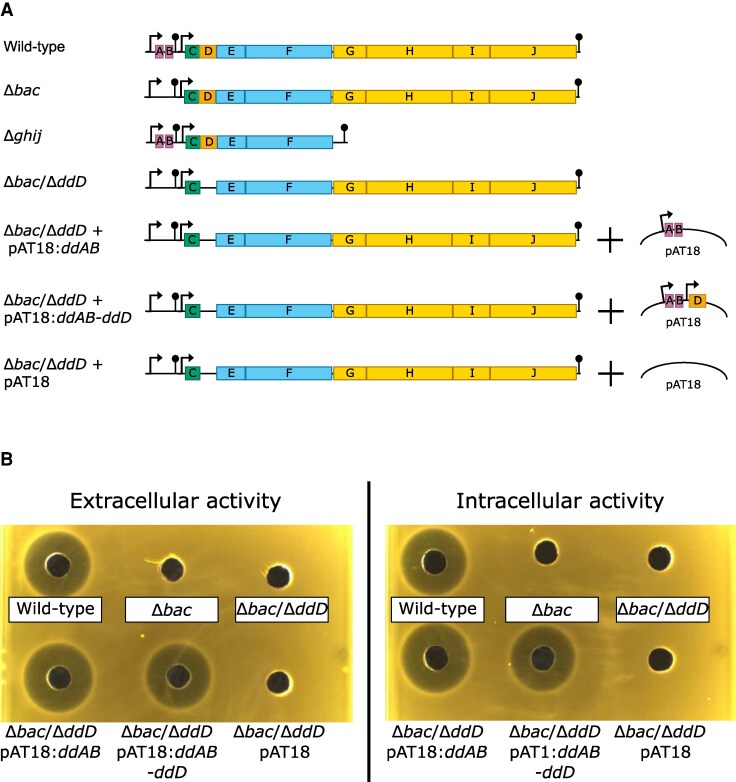
Schematic representation of *E. faecalis* 14 mutants showing their genotypes and antibacterial activities against *L. innocua*. A) Schematic representation of the deletion mutants. Color coding indicates the functional grouping of genes: purple, bacteriocin precursors; blue, PH domain–based transporters; yellow, ABC transporters; green (ddC) and orange (ddD), genes of unknown function. B) The antibacterial activity of the supernatant (left) and cellular fraction (right) of the different mutant strains against *L. innocua* ATCC 33090. In both cases, 50 μL samples were applied to an overlay of the target strain. The presence of an inhibition zone indicates susceptibility to the bacteriocin EntDD14. Data are representative of three independent experiments.

In silico analyses suggested that DdD may be involved in transport. This led us to hypothesize that DdD could direct EntDD14 toward one of the two known transport systems: DdEF or the DdGHIJ ABC transporter. To test this hypothesis, we attempted to delete the DdD coding sequence (CDS) and analyze the resulting phenotype. We carried out deletion by homologous recombination using the thermosensitive vector pLT06 (72). Although the initial recombination steps were successful, the final deletion mutant could not be obtained in the wild-type strain. This suggests that the absence of DdD might be lethal. We hypothesized that the loss of DdD could impair EntDD14 export, resulting in the intracellular accumulation of the bacteriocin and subsequent toxicity. To test this hypothesis, we repeated the deletion in a *Δbac* background in which both bacteriocin structural genes (*ddA* and *ddB*) had been deleted (Fig. 2A). In this context, the deletion of *ddD* was easily achieved, confirming that the absence of *ddD* is detrimental only when the EntDD14 coding genes are present. Since bacteriocin production was necessary to evaluate transport with or without *ddD*, we complemented the *Δbac/ΔddD* mutant with the *ddAB* operon carried by the plasmid pAT18:*ddAB*.

*Trans* complementation was also performed with both *ddAB* and *ddD* (by transferring the plasmid pAT18:*ddAB*-*ddD*) to assess potential phenotypic differences linked to the absence of DdD's production. It is worth noting that previous transcriptomic analyses of the *Δbac* mutant revealed significant downregulation of the *ddCDEFGHIJ* operon, despite its physical separation from *ddAB* (65). This indicates regulatory coupling between the structural bacteriocin genes and the downstream operon. Taken together, these results suggest that the expression of *ddD* under its native regulatory context would likely be reduced in the absence of *ddAB*. Consequently, complementation with *ddD* alone was not pursued, since expression of *ddD* under its native promoter would likely be insufficient in the absence of *ddAB*. Artificial overexpression from a heterologous promoter was not considered, as this would not reflect the locus's physiological regulatory context. An additional control with the empty vector pAT18 was included to rule out phenotypic changes associated with the vector. All *E. faecalis* mutant strains were successfully constructed, and their genetic backgrounds were confirmed by the PCR and DNA sequencing. The construction strategy is shown in Fig. 2A. The antibacterial activity of the constructed strains was evaluated using an agar diffusion assay with *Listeria innocua* ATCC 33090 as the indicator strain (Fig. 2B). To assess the potential role of DdD in the EntDD14 export, we analyzed both the extracellular (cell-free supernatant) and intracellular fractions. As expected, the positive and negative controls produced the anticipated results. The *Δbac*/*ΔddD* double mutant showed no inhibitory activity, which is consistent with the absence of the bacteriocin structural genes. In contrast, complementation with the *ddAB* operon restored antibacterial activity in both the extracellular and intracellular fractions. However, complementation with the empty vector did not have this effect. Halo size measurements showed no significant difference between the wild type and both *Δbac*/*ΔddD* complemented strains (Fig. S2). These results demonstrate that DdD is not required for EntDD14 synthesis, export, or activity, despite the in silico predictions suggesting a transport-related role.

### The DdD protein is involved in the resistance to EntDD14

**Figure 3:**
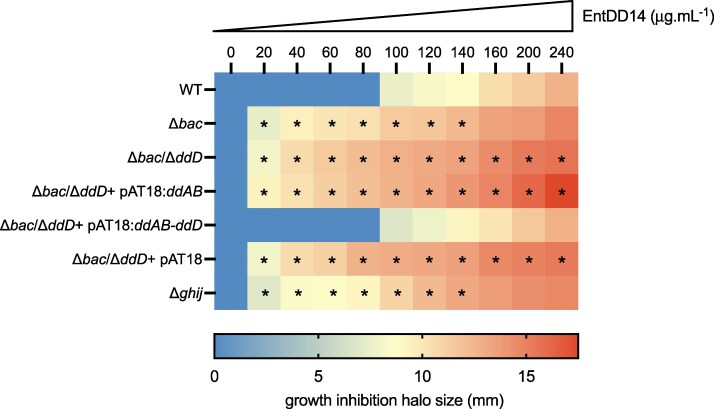
Heat map resistance profile of *E. faecalis* 14 and its mutant strains against external EntDD14. All strains were cultivated overnight and then spread onto GM17 agar. Increasing concentrations of pure EntDD14 were spotted onto an overlay of the target strain. After incubation for 24 h at 37 °C, the inhibition halos were measured. MIC values correspond to the lowest concentration producing a visible inhibition halo in the agar diffusion assay (represented by blue square). An asterisk (*) indicates results that differ significantly from those of the wild-type strain at the same EntDD14 concentration (*P* < 0.0001, one-way ANOVA). The data represent the mean of at least three independent experiments.

The lack of DdD involvement in EntDD14 synthesis or transport suggested that its function may lie elsewhere within the bacteriocin system. The fact that DdD deletion was only possible in the *Δbac* background strongly suggests that DdD is necessary for bacterial survival in the presence of EntDD14. Furthermore, in silico analyses indicated that DdD may interact with other proteins, including EntDD14 itself. This suggests a potential role in self-immunity (Fig. 1 and Table 1). To test this, we examined the intrinsic resistance of different mutants to purified EntDD14 (Fig. 3). As previously reported, the *ΔddGHIJ* mutant displayed lower resistance than the wild-type strain. The minimum inhibitory concentration (MIC) threshold, as determined by agar diffusion, decreased from 120 µg/mL for the wild-type strain to 60 µg/mL for the mutant (15). The *Δbac* mutant showed even greater sensitivity, with an MIC of 20–40 µg/mL. The *Δbac/ΔddD* double mutant had an MIC similar to that of the Δ*bac* strain. Interestingly, complementing the *Δbac/ΔddD* strain with *ddAB* alone decreased resistance further, resulting in an MIC below the lowest concentration tested (20 µg/mL). This suggests that intracellular production of EntDD14 in the absence of DdD may slightly decrease the strain's overall resistance. This may explain why the noncomplemented double mutant was not more sensitive than the *Δbac* strain, whereas the *ddAB*-complemented derivative was. Both the *Δbac/ΔddD* strain (with and without *ddAB* complementation) displayed significantly reduced resistance compared with the wild type at all tested concentrations.

**Figure 4:**
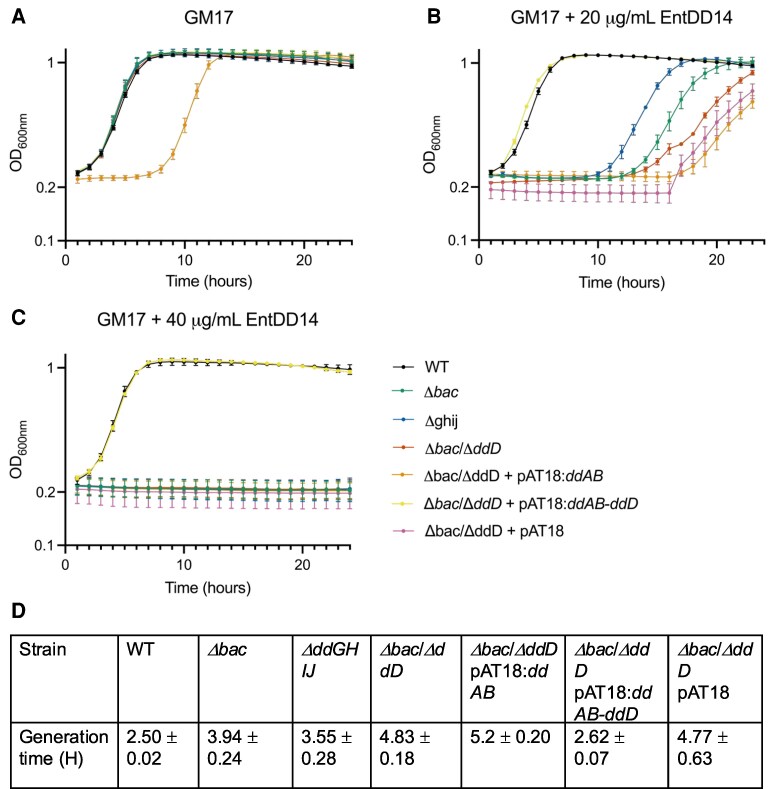
Growth curves of *E. faecalis* 14 and its mutant strains in GM17 broth (A) and in the presence of EntDD14 at 20 μg/mL (B) or 40 μg/mL (C). D) The generation time of each tested strain during growth in the presence of EntDD14 at 20 μg/mL. Calculated generation times for all mutant strains showed significant differences compared with the wild-type strain (*P* < 0.0001, one-way ANOVA). The data represent the mean of at least three independent experiments. Error bars and the ± sign represent the SD of the biological replicates.

To further investigate the role of EntDD14 in bacteriocin resistance, growth kinetics were monitored in the presence of 20 or 40 µg/mL of EntDD14 (Fig. 4). In the absence of added EntDD14, no significant growth differences were observed among most strains except for the Δ*bac*/Δ*ddD* strain complemented with *ddAB*, which displayed a growth delay of ∼8 h. This delay was absent in the noncomplemented *Δbac/ΔddD* strain; therefore, the phenotype is presumably attributable to intracellular EntDD14 production. Complementation with both *ddAB* and *ddD* restored wild-type growth, confirming that the deleterious effect of EntDD14 is specifically due to the absence of *ddD*. The wild type grew normally in the presence of EntDD14 at 20 µg/mL; however, the *ΔddGHIJ* mutant displayed a lag of ∼10 h, which is consistent with previous findings (15). A longer delay of 13 h was observed for the *Δbac* strain. The *Δbac/ΔddD* mutant exhibited a similar lag to that of the single mutant. Interestingly, the presence of the bacteriocin structural genes in *the Δbac*/*ΔddD* + *ddAB* strain resulted in an even longer lag of ∼17 h. This demonstrated that the loss of DdD production markedly increases sensitivity to extracellular EntDD14 and that the delay observed in the *Δbac*/*ΔddD* mutant was not solely the result of the *Δbac* mutation. In the presence of 40 µg/mL of EntDD14, all mutant strains failed to grow except the double-complemented *Δbac*/*ΔddD* strain, which exhibited fully recovered wild-type growth (Fig. 4C). Consistent with these observations, calculation of the generation times for growth in the presence of EntD14 at 20 µg/mL (Fig. 4D) confirmed that wild-type cells maintained a generation time similar to control conditions. In contrast, all mutant strains exhibited significant growth defects. The *ΔddGHIJ* strain displayed a moderate increase in generation time, consistent with its known partial contribution to resistance, while the *Δbac* mutant showed an even greater increase. Interestingly, although the *Δbac*/*ΔddD* double mutant did not exhibit a longer lag phase at the onset of growth compared with the *Δbac* single mutant, it nevertheless showed a slower growth rate, indicating that deletion of the *ddD* gene further exacerbates sensitivity to EntDD14. Complementation of this background with the *ddAB* genes alone did not substantially alter the generation time, although it did delay the onset of growth by ∼2 h (Fig. 4B). In contrast, the double complementation with both the *ddAB* and *ddD* genes fully rescued the phenotype, increasing both the onset of growth and the generation time up to the wild-type levels. To rule out the possibility of rapid adaptive mutation, cultures taken at the end of the lag phase were reinoculated and exhibited identical growth kinetics. This indicates that the observed delay is not the result of compensatory evolution (Fig. S3).

### Predicted structures of DdD show a potential interaction with EntDD14

**Figure 5:**
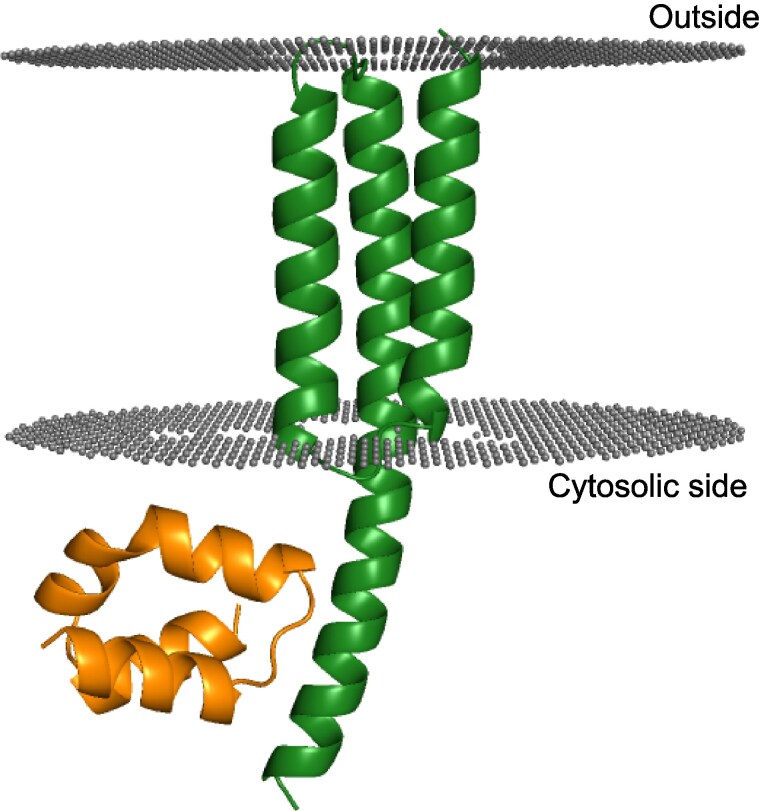
Model of the membrane orientation for the AF3 DdD–DD14A interaction complex based on the 1D deep learning predictive algorithm DeepTMHMM 1.0 and the 3D prediction from the programs TmDet 4 and PPM 3.0. DdD backbone in green, DD14A in brown.

To investigate the structural basis of the DdD–DD14A interaction, a 3D model of the complex was generated using AF3. The predicted structure revealed a stable association between the transmembrane region of DdD and the cytosolic domain of DD14A with a good confidence score (average pLDDT score = 72.6, in the corresponding range for confident models). The AF3 complex was then oriented relative to a lipid bilayer using PPM 3.0 and the membrane topology previously established (Fig. 1). The obtained model is consistent with DdD acting as an integral membrane anchor and DD14A associating with its cytosolic face (Fig. 5). This integrated modeling approach supports a plausible membrane-associated interaction between DdD and DD14A.

## Discussion

In this study, we present the first functional characterization of DdD within the *E. faecalis* EntDD14 gene cluster. Our findings demonstrate that DdD plays a pivotal role in self-immunity, rather than in bacteriocin synthesis or transport. Although in silico predictions suggested a possible role for DdD in membrane transport, our genetic and phenotypic analyses revealed that the protein is dispensable for EntDD14 production and secretion (Figs. 1 and 2). Because deleting *ddD* compromised the viability of *E. faecalis* and prevented recovery of the mutant in a wild-type background, we hypothesized that DdD plays an immunity role. This hypothesis was confirmed by resistance assays of different mutant strains (Figs. 3 and 4). The immunity mechanisms of LBB are poorly understood, but they typically involve ABC transporters in combination with other unidentified factors. For instance, an ABC transporter contributes to protection in aureocin A53, yet additional proteins are necessary for complete resistance (56). Similarly, immunity to lacticin Q requires a multidrug transporter, though unidentified components are also necessary for complete self-resistance (73). Our results identify DdD as a novel accessory factor in this category that complements the activity of the ABC transporter DdGHIJ. Previous studies have shown that DdGHIJ mediates both export and resistance (16, 65). Since the knockout strain of the *ddD* gene produced stronger effects than the knockout of the *ddghij* genes, we propose that the DdD protein provides a more effective contribution to immunity against EntDD14. This pattern, in which the accessory factor plays a more critical role than the ABC transporter, has been observed in other bacteriocin immunity mechanisms (56, 73–77). In silico analyses further support this interpretation. Structural predictions indicate that DdD is a cationic protein, which is associated with the membrane via multiple putative transmembrane regions (78–82). Moreover, a prominent cytoplasmic helix was predicted and docking models suggest that this helix binds to EntDD14, specifically to the DdA monomer. This implies that DdD may function by binding directly to the bacteriocin prior to its secretion. This would lower the bacteriocin's concentration at the membrane and prevent premature toxicity. Due to the absence of conserved immunity motifs and the lack of direct homology to characterized proteins, DdD may stabilize transport systems or sequester EntDD14 precursors prior to secretion. Additional biochemical assays, such as co-immunoprecipitation or cross-linking experiments, are needed to confirm direct interaction between DdD and EntDD14. This model is consistent with the growth defects observed in *ΔddD* strains complemented with *ddAB*, in which intracellular bacteriocin synthesis occurs without DdD protection. It also explains why the deletion of *ddD* is lethal in a background that carries the EntDD14 structural genes, yet tolerable in the *Δbac* mutant strain that does not produce bacteriocin endogenously. However, in the *Δbac/ΔddD* strain, complementation with the structural genes was feasible without an inducible promoter. This indicates that plasmid-driven expression differs from native chromosomal regulation. In the wild-type background, the *ddAB* genes are integrated within the genome and expressed under the control of a tightly regulated promoter, likely ensuring synchronized production, transport, and immunity of bacteriocins. Under these conditions, DdD appears to be essential for counterbalancing bacteriocin self-production, which explains why it could not be deleted. In contrast, the level of expression, timing, and regulatory coupling of plasmid-expressed *ddAB* with the downstream *ddCDEFGHIJ* operon may differ. These potential differences could reduce bacteriocin toxicity, enabling viability despite the absence of DdD.

Importantly, this condition remains physiologically suboptimal, as evidenced by the substantial growth delay observed with *ddAB* complementation alone (Figs. 3 and 4). Furthermore, the *Δbac* mutant exhibits partial sensitivity to EntDD14, a sensitivity that is increased by the deletion of *ddD*. This supports the idea that DdD specifically contributes to self-immunity rather than to bacteriocin production or secretion.

Furthermore, the *ddD* deletion was achieved in-frame and in a markerless manner to avoid polar effects on downstream genes. The functional restoration of bacteriocin export in the *ddAB*-only complemented strain confirms that both export systems, encoded by *ddEF* and *ddGHIJ*, respectively, remain active (Fig. 2). Although adaptive mutations cannot be completely excluded, the full restoration of normal growth and resistance following double complementation with *ddAB* and *ddD* strongly suggests that the observed phenotype is specifically due to the absence of DdD rather than secondary genetic changes (Figs. 3 and 4).

This study expands the repertoire of known immunity factors and emphasizes the significance of accessory proteins beyond canonical ABC transporters. Future biochemical studies testing the physical interactions between DdD and EntDD14 will be essential to confirm this mechanism and further elucidate the role of DdD in the unique immunity architecture of LBBs. Overall, the discovery of DdD as a critical EntDD14 immunity determinant underscores the intricacy of self-defense systems in LBB producers. Since these peptides are synthesized in their active form, the immune system must coordinate closely with the synthesis and export processes (48). Recent studies have shown that such coordination often depends on multilayered regulatory mechanisms, including posttranscriptional control of mRNA stability (83), as well as the presence of putative regulatory genes within bacteriocin operons (84). Nevertheless, the molecular basis of regulation in LBB bacteriocins remains poorly understood (49, 85, 86). Further studies are crucial to defining the control layers that govern EntDD14 expression.

## Materials and methods

### Bacterial strains and growth conditions

The bacterial strains, plasmids, and oligonucleotides used in this study are listed in Table 2. *Enterococcus faecalis* cultures were grown in M17 medium (Sigma-Aldrich, United States) supplemented with 0.5% glucose at 37 °C (GM17), and *L. innocua* ATCC 33090 cultures were grown in brain heart infusion (BHI; Sigma-Aldrich) at the same temperature. *Escherichia coli* strains were grown in Luria–Bertani (Sigma-Aldrich) broth at 37 °C with shaking at 160 rpm. When necessary, the media were supplemented with erythromycin (Em) at a concentration of 150 μg/mL or chloramphenicol (Cm) at a concentration of 30 μg/mL.

**Table 2:** List of the bacteria used in this work.

Bacteria		
Strain	Characteristics	Ref.
*Escherichia coli*		
XL1-Blue	*recA1 endA1 gyrA96 thi-1 hsdR17 supE44 relA1 Lac,* carrying [F′ *proAB lacI* qZΔM15Tn*10* (TetR)]	Stratagene
JM109	*recA1*. *endAl*, gyrA96, *thi*. *hsdR17*, *supE44*, *relA1*, I-, A(*iac*-*proAB*), carrying [F′, *traD36*, *proAB*, *iacIqZAM15*]	(87)
*Enterococcus faecalis*		
14	Wild-type strain isolated from meconium	(60)
14 *Δbac*	Strain lacking the *ddAB* genes	(65)
14 Δ*ddGHIJ*	Strain lacking the *ddGHIJ* genes	(15)
14 Δ*ddAB/*Δ*ddD*	Strain lacking the *ddAB* and *ddD* genes	This study
14 Δ*ddAB/*Δ*ddD* + pAT18:*ddAB*	Strain lacking the *ddAB* and *ddD* genes and carrying the pAT18:*ddAB* plasmid	This study
14 Δ*ddAB/*Δ*ddD* + pAT18:*ddAB*-ddD	Strain lacking the *ddAB* and *ddD* genes and carrying the pAT18:*ddAB-ddD* plasmid	This study
*Listeria innocua*		
ATCC 33090		(88)

Plasmid		
Name	Characteristics	Ref.
pLT06	Carrying: *lacZ*, P-*pheS* from plasmid pCJK47, *cat* gene from plasmid pGB354, and *orfB*, *orfC*, *repA* (*Ts*), *orfD* from plasmid pCASPER	(72)
pLT06:*ΔddD*	Derivative of pLT06 by cloning of a 1,983-bp DNA fragment harboring the flanking regions of the *ddD* gene	This study
pAT18	*Erm^R^*, Tra−, Mob+, *Iac*Za+, *ori*R of plasmid pUC, *ori*R from plasmid pAMβI, and MCS of plasmid pUCl8	(89)
pAT18:*ddAB*	pAT18 derivative carrying a 640-bp DNA fragment from *E. faecalis* 14 containing the *ddAB* genes	(65)
pAT18:*ddAB-ddD*	pAT18 derivative carrying a 640-bp DNA fragment from *E. faecalis* 14 containing the *ddAB* genes and a 579-bp DNA fragment from *E. faecalis* 14 containing the *ddD* gene and its natural promoter region	This study

### Construction of the *E. faecalis* 14 Δ*bac*/Δ*ddD* mutant strain

The *ddD* gene was deleted from the chromosome of *E. faecalis* 14 through successive recombination events using the pLT06 plasmid (72). A detailed description of the protocol can be found in the work of Pérez-Ramos et al. (18). In brief, the genomic regions flanking the *ddD* coding sequence were amplified by PCR, digested with the pLT06 vector, and ligated to create the deletion construct. After transformation into *Es. coli* XL-1 Blue, the recombinant plasmids were confirmed by PCR, restriction digestion, and DNA sequencing analyses. After the transformation into *Es. coli* XL-1 Blue, the recombinant plasmids were confirmed by PCR, restriction digestion and DNA sequencing. The oligonucleotides used in these steps are listed in Table 3. The validated construct was introduced into the *E. faecalis* 14 *Δbac* strain by electroporation. The first recombination event, chromosomal integration of the plasmid, was induced by incubating the strain at 42 °C for 4 h in the presence of chloramphenicol (Cm), and integration was verified by PCR. Positive clones were subjected to a second recombination event by shifting the growth temperature to 30 °C without antibiotic selection. This allowed for plasmid excision. Candidate clones were screened for deletion of the target locus by PCR and verified gene cluster deletions by DNA sequencing of the flanking regions.

**Table 3:** List of oligonucleotides used in this work.

Oligonucleotide	Sequence 5′-3′	Utilization	Amplicon size (pb)
*ddD* 1F-PstI	ATTAAACTGCAGTCTCATTTGTTGAGCTATTT	Amplification of the *ddD* upstream fragment	1,036
*ddD* 2R-Stop	CATTCACTAGGATCCTTAGACTTAACTCATTTATAACATCTCCT		
*ddD* 3F-Stop	TAAGTCTAAGGATCCTAGTGAATGAAAAGAGAAATTTTTCAATG	Amplification of the *ddD* downstream fragment	1,019
*ddD* 4R-NcoI	ATTAAACCATGGAATCCAATGAAGATAACAAG		
*ddD* 5F	CTTTATTAAACATGAGATATTGAAG	Outer primer; verification of the plasmid integration	—
*ddD* 6R	CAGGAATACTATTAAATAGATATTC		
*oriF*	CAATAATCGCATCCGATTGCA	Verification of cloning in the pLT06 plasmid	2,214
Ks05R	CCTATTATACCATATTTTGGAC		
P-*ddD*-F	CAACATGATGGTACCGAGCTTTTTTATATTATATTAAATTTTGAAAAAGACTG	Cloning of the promoter region of *ddD* gene in the pAT18 plasmid	246
P-*ddD*-R	TCAAACTCATTTTTACCTCCTATAATAGACAATTG		
*ddD*-comp-F	GGAGGTAAAAATGAGTTTGAATGTAATTGTG	Cloning of the *ddF* gene in the pAT18 plasmid	303
*ddD*-comp-R	CAGCTATGACCATGATTACGAATAATAAATAATTATGATGCATTACAAATTC		
*ddB*-inside-F	CCCATCCTTCTCCAATAAATTGC	Verification in the pAT18:*ddAB* plasmid	—
*ddD*-R	CCCGGGAATAATAAATAATTATGATGCATTA		
pAT18-R	GATTCATTAATGCAGCTGGCACGA		

### Double complementation of the *E. feacalis* 14 Δ*bac*/Δ*ddD* mutant strain

The *ddD* gene, together with its native transcriptional promoter, was cloned into the pAT18:*ddAB* plasmid to complement the *Δbac/ΔddD* mutant strain (65, 89). The protocol was followed as previously described (18) except for the vector assembly step. In this case, the *ddD* gene and the promoter region of the *ddC-J* operon were amplified by PCR (Table 3) and cloned into the pAT18:*ddAB* plasmid, which was previously digested with *SacI* and *EcoRI*, using the NEBuilder HiFi DNA Assembly Kit (New England Biolabs, United States). The recombinant plasmids were verified by PCR, restriction digestion, and DNA sequencing. The validated construct was introduced into the *E. faecalis* 14 *Δbac/ΔddD* strain by electroporation. In parallel, electroporation with the pAT18:*ddAB* or the empty pAT18 plasmids was performed as a control.

### Antibacterial activity assays

The antimicrobial activity of the wild-type *E. faecalis* 14 and its isogenic mutant strains against the indicator strain *L. innocua* ATCC 33090 was evaluated using a well-diffusion assay, as previously described (90). In brief, overnight cultures of different *E. faecalis* strains were harvested and centrifuged to separate the pellets from the supernatant. The pellets were washed twice with GM17 medium prior to lysis to minimize contamination by extracellular bacteriocin and the intracellular components were extracted using a French press. The supernatants were then centrifuged again and filtered to obtain cell-free preparations. For the assay, an overnight culture of *L. innocua* was diluted to 1% (v/v) in soft BHI agar (1% agar) and overlaid on standard BHI agar plates (1.5% agar). Then, wells were generated into the agar, and 50 μL of either culture supernatant or intracellular extract was added to each well. The plates were then incubated overnight at 37 °C, and the inhibition zones surrounding the wells were recorded.

### Sensitivity of the wild type and the engineered variants to extracellular EntDD14

The parental strain and the mutant's behavior were tested for self-resistance against their own bacteriocin, when it was supplied extracellularly. As previously reported, pure EntDD14, which was chemically synthesized (52), was used in these assays following the previously described protocol (15). In brief, exponential-phase cultures of each strain were adjusted to 10^6^ CFU/mL and spread onto GM17 agar plates with sterile swabs. Then, 10 µL of purified EntDD14 solution at various concentrations was spotted onto the plates. After overnight incubation at 37 °C, the inhibition halos were measured. The MIC was defined as the lowest concentration of EntDD14 that produced a clearly visible zone of inhibition (i.e. a complete absence of visible bacterial growth) under standardized assay conditions. In parallel, we assessed bacterial growth in a liquid medium in the presence of EntDD14. Strains were inoculated into GM17 broth, with or without EntDD14 (at concentrations of 20 or 40 µg/mL), in 96-well microplates. The cultures were initiated from overnight precultures, adjusted to an initial OD_600 nm_ of 0.2, and monitored over 24 h at 37 °C. Optical density was recorded every 15 min at OD_600 nm_ using a SpectraMax i3 spectrophotometer (Molecular Devices, San Jose, CA).

### Structure predictions and annotations

DdD structures were predicted using AF3 with the corresponding FASTA sequence as input (91). Protein structure and function predictions were also performed using the D-I-TASSER method (92). The DeepTMHMM server v.1.0 (https://services.healthtech.dtu.dk/services/DeepTMHMM-1.0, accessed 2025 August 1) was used to predict transmembrane topology, supported by program Tmbed (https://github.com/BernhoferM/TMbed) and membranes were added to the model according to this prediction (93, 94). Sequence similarity searches were performed using BLAST (National Center for Biotechnology Information, BLAST-NCBI: https://blast.ncbi.nlm.nih.gov/Blast.cgi, accessed 2025 August 1) against the Clustered NR database. The NetGO v4.0 server (https://dmiip.sjtu.edu.cn/ng4.0/, accessed 2025 August 2) was used to identify GO terms for the DdD protein sequence (95). The structure of the DdD–DdA interaction complex was predicted using AF3, while the orientation of the AF3 complex in a lipid bilayer was realized using PPM 3.0 and, previously, DeepTMHMM and Tmbed analysis (96).

## Supplementary Material

pgag192_Supplementary_Data

## Data Availability

All study data are included in the article.
