# Supplementary material for: The DdD protein confers intracellular and extracellular immunity to the leaderless bacteriocin enterocin DD14
Source: PNAS Nexus. 2026 Jun 12;5(6):pgag192. doi: 10.1093/pnasnexus/pgag192 (PMC13262744; doi:10.1093/pnasnexus/pgag192)
Supplement: pgag192_Supplementary_Data [file pgag192_supplementary_data.docx]

Supplementary Data

**The DdD Protein Confers Intracellular and Extracellular Immunity to the Leaderless Bacteriocin Enterocin DD14**

Clémence Cochard^1^, Adrián Pérez-Ramos^1Φ^, Mari Luz Mohedano^2^, Mario García de Lacoba^3^ Paloma López^2^, Djamel Drider^1*^

^1^ Unité Mixte de Recherche (UMR) Transfrontalière BioEcoAgro INRAE 1158, Université de Lille 59000, Lille, France

^2^ Departamento de Biotecnología, Centro de Investigaciones Biológicas Margarita Salas (CIB, CSIC), 28040 Madrid, Spain

^3^Departamento de Bioinformatics y Biostatistics. Centro de Investigaciones Biológicas Margarita Salas (CIB, CSIC), 28040 Madrid, Spain

Φ Current address : Centro de Biología Molecular
Severo Ochoa, CSIC-UAM, Campus de la Universidad Autónoma de Madrid, 28049 Madrid, Spain.

Correspondence: D. Drider ([djamel.drider@univ-lille.fr](mailto:djamel.drider@univ-lille.fr))

DdD 1 MSLNVIVLLALLAVQTWLIPRLNNKFLLLIVPSIFVVLSIYIYIENLSLL 50

||:.|||||.||||||||:|:||||.||.::|.|||.||:|::.|.||||

L50F 1 MSVEVIVLLGLLAVQTWLMPKLNNKHLLFLIPGIFVALSLYVFREKLSLL 50

DdD 51 IIIGLMFGFFIYYMAGLTQWDRINKERQKKYLKEKFFNEK 90

|::|||.||||||||||:|||||..|:|||:|||||||||

L50F 51 IVMGLMLGFFIYYMAGLSQWDRIKNEKQKKHLKEKFFNEK 90

**Supplementary Figure 1.** Sequence comparison between DdD proteins from E. faecalis 14 and E. faecium L50F. Sequences were aligned and compared using the Emboss Needle software.

| WT | Δbac/ΔddD + pAT18-*ddAB* | Δbac/ΔddD + pAT18-*ddAB-ddD* |
| --- | --- | --- |
| 2.87 ± 0.12 | 2.77 ± 0.15 | 2.73 ± 0.06 |

**Supplementary Figure 2.** Halo size measurement of the antibacterial activity of the supernatant of the different mutant strains against *L. innocua* ATCC 33090. Fifty μL samples were applied to an overlay of the target strain. After 24 hours of incubation, growth inhibition halo sizes were measured. Data are representative of three independent experiments.

**Supplementary Figure 3.** Growth curves of E. faecalis 14 and its mutant strains in GM17 broth and in the presence of 20 μg/mL EntDD14. The different strains were first cultured for 24 hours (A) before being reinoculated to monitor regrowth (B). The data represent the mean of at least three independent experiments.
